# Robotic versus Laparoscopic Surgery for Spleen-Preserving Distal Pancreatectomies: Systematic Review and Meta-Analysis

**DOI:** 10.3390/jpm11060552

**Published:** 2021-06-13

**Authors:** Gianluca Rompianesi, Roberto Montalti, Luisa Ambrosio, Roberto Ivan Troisi

**Affiliations:** Division of Hepato-Bilio-Pancreatic, Minimally Invasive and Robotic Surgery, Department of Clinical Medicine and Surgery, Federico II University Hospital, Via S.Pansini 5, 80131 Naples, Italy; gianluca.rompianesi@unina.it (G.R.); luisa.ambrosio@unina.it (L.A.); roberto.troisi@unina.it (R.I.T.)

**Keywords:** robotic distal pancreatectomy, laparoscopic distal pancreatectomy, spleen-preserving distal pancreatectomy, minimally-invasive distal pancreatectomy, systematic review, meta-analysis

## Abstract

Background: When oncologically feasible, avoiding unnecessary splenectomies prevents patients who are undergoing distal pancreatectomy (DP) from facing significant thromboembolic and infective risks. Methods: A systematic search of MEDLINE, Embase, and Web Of Science identified 11 studies reporting outcomes of 323 patients undergoing intended spleen-preserving minimally invasive robotic DP (SP-RADP) and 362 laparoscopic DP (SP-LADP) in order to compare the spleen preservation rates of the two techniques. The risk of bias was evaluated according to the Newcastle–Ottawa Scale. Results: SP-RADP showed superior results over the laparoscopic approach, with an inferior spleen preservation failure risk difference (RD) of 0.24 (95% CI 0.15, 0.33), reduced open conversion rate (RD of −0.05 (95% CI −0.09, −0.01)), reduced blood loss (mean difference of −138 mL (95% CI −205, −71)), and mean difference in hospital length of stay of −1.5 days (95% CI −2.8, −0.2), with similar operative time, clinically relevant postoperative pancreatic fistula (ISGPS grade B/C), and Clavien–Dindo grade ≥3 postoperative complications. Conclusion: Both SP-RADP and SP-LADP proved to be safe and effective procedures, with minimal perioperative mortality and low postoperative morbidity. The robotic approach proved to be superior to the laparoscopic approach in terms of spleen preservation rate, intraoperative blood loss, and hospital length of stay.

## 1. Introduction

The decision on preserving the spleen when performing a distal pancreatectomy (DP) is usually based on the balance between achieving an adequate oncological clearance and avoiding complications related to asplenia. Spleen-preserving DP has therefore been mainly reserved for surgeries performed for benign indications or to excise lesions with a low malignant potential. With the advent of minimally invasive surgery, in the early 1990s, surgeons around the world started to explore the potential of the laparoscopic approach in pancreatic surgery [1,2] and, almost a decade later, of the robotic-assisted technique [3]. Minimally invasive pancreatic surgery has been progressively gaining widespread popularity, and advancements in surgical skills have removed most of the technical restrictions, allowing the safe and effective execution of complex procedures, including laparoscopic spleen-preserving distal pancreatectomy (SP-LADP) [4] and robot-assisted spleen-preserving distal pancreatectomy (SP-RADP) [5].

This systematic review and meta-analysis aims to summarize all of the available evidence regarding spleen-preserving DP and compare results and outcomes of minimally invasive SP-RADP and SP-LADP techniques.

## 2. Materials and Methods

This systematic review and meta-analysis was conducted in accordance with the preferred reporting items for systematic reviews and meta-analyses (PRISMA 2020 Statement [6]) and was registered on PROSPERO (CRD42021239032).

### 2.1. Search Strategy

MEDLINE, Embase, and Web Of Science electronic databases were searched using the following terms: “pancrea*” AND “robot*” AND “laparoscop*” AND “sple*”. The last search was run on 1 February 2021 with no language or publication status restrictions. Additional potentially relevant studies were identified from the reference lists of selected studies.

### 2.2. Study Selection

For inclusion, studies had to (1) include patients undergoing DP for any disease; (2) include procedures performed robotically and laparoscopically; and (3) report data on patients undergoing DP with the intent of preserving the spleen. Case reports, reviews, and communications, as well as non-human studies, were excluded. Two reviewers (G.R. and L.A.) independently screened the results of the electronic search at title and abstract levels. The full texts of the selected references were also retrieved for further analysis and data extraction. When duplicate reports from the same study were identified, only the most recent publication was included.

### 2.3. Data Extraction and Quality Assessment

Two reviewers (G.R. and L.A.) extracted data from each selected study regarding the first author; publication year; country of origin; study design; number of patients undergoing SP-RADP and SP-LADP; patients characteristics (age, sex, body mass index (BMI)); underlying disease requiring DP; American Society of Anesthesiologists (ASA) score; tumor size; conversion rate; blood loss; pancreatic stump closure technique; splenic vessel preservation and technique (Warshaw vs. Kimura); blood transfusion requirement; length of surgery; data on postoperative morbidity, including prevalence and grading of the clinical severity of postoperative pancreatic fistula (POPF) according to the ISGPS definition [7]; complications and grading according to the Clavien–Dindo classification [8]; re-operation rate; length of stay (LOS); mortality; and length of follow-up. The quality and risk of bias of each included study was evaluated independently by two reviewers (G.R. and L.A.) according to the Newcastle–Ottawa Scale for evaluating the quality of non-randomized studies in meta-analyses [9]. The level of evidence was rated according to the Grading of Recommendations, Assessment, Development and Evaluations (GRADE) system [10]. Any disagreement was resolved through discussion in order to reach consensus across the study team.

### 2.4. Statistical Analysis and Data Synthesis

The primary outcome was the spleen preservation failure rate. Secondary outcomes included intraoperative blood loss, operative time, prevalence of clinically relevant POPF (grade B/C), prevalence of postoperative complications (Clavien–Dindo [8] grades ≥3), hospital LOS, and mortality. For the analysis, values expressed as median (range) were converted to average ± standard deviation using Wan’s method [11]. To pool proportions, we used random-effects or fixed-effect modelling according to the DerSimonian and Laird method [12,13] to take into account heterogeneity. The presence of heterogeneity among the studies was assessed using Cochran’s Q test and quantified with the I^2^ inconsistency index, with 25, 50, and 75% considered as thresholds for low, moderate, and high statistical heterogeneity, respectively. Heterogeneity was evaluated by sensitivity analysis [14]. Statistical analyses were performed using Review Manager version 5.3.

## 3. Results

### 3.1. Studies Selection

**Figure 1:**
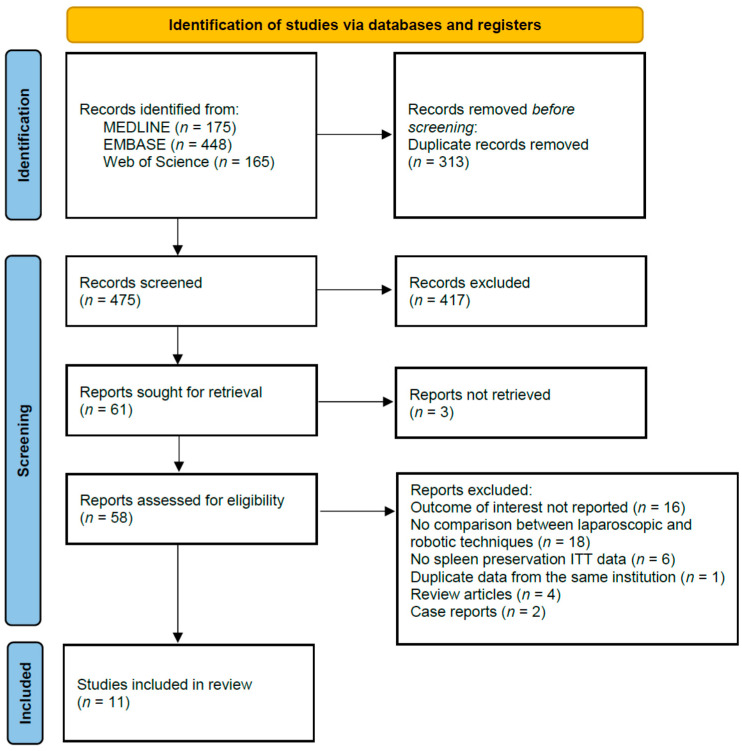
PRISMA flow diagram. ITT: intention-to-treat.

Eleven studies met the inclusion criteria and were included in the systematic review and meta-analysis [15,16,17,18,19,20,21,22,23,24,25] (Figure 1).

### 3.2. Studies Characteristics

The characteristics of the selected studies are reported in Table 1. A total of 323 patients undergoing SP-RADP and 362 patients undergoing SP-LADP were included in this meta-analysis. Eight included series (72.7%) were retrospective cohort studies [16,17,18,21,22,23,24,25], two were matched cohort studies (18.2%) [15,19], and one was a case-control study (9.1%) [20]. The reported median follow-up was 27 months (range 6.5–47) for SP-RADP and 33.5 months (range 32–75.5) for SP-LADP. The most frequent indications for surgery were neuroendocrine tumors (NET) in 61 SP-RADP and 52 SP-LADP, mucinous cystic neoplasms in 37 SP-RADP and 28 SP-LADP, intraductal papillary mucinous neoplasms (IPMN) in 15 SP-RADP and 28 SP-LADP, and pseudopapillary tumors in 18 SP-RADP and 17 SP-LADP.

### 3.3. Quality Assessment and Publication Bias

The results of the quality assessment of the 11 included studies according to the guidelines of the Newcastle–Ottawa Scale are reported in Table 1.

### 3.4. Spleen Preservation Rate

**Figure 2:**
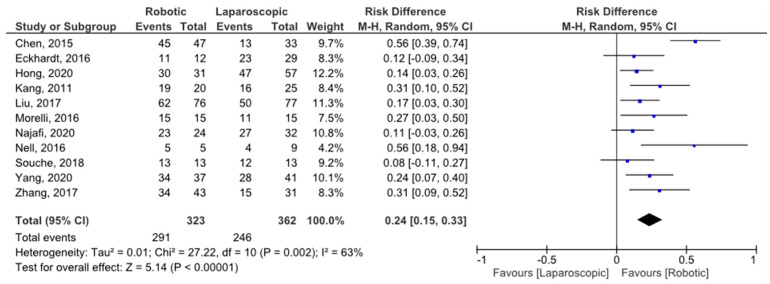
Spleen preservation rate forest plot.

All selected studies reported the number of procedures intended to be spleen preserving and the spleen preservation failure rate for both the robotic and laparoscopic techniques. The risk difference (RD) of spleen preservation failures was 0.24 (95% CI 0.15, 0.33), favoring the robotic approach and with moderate heterogeneity (I^2^ = 63%) (Figure 2). Heterogeneity was evaluated by sensitivity analysis, and the results are summarized in Table 2.

### 3.5. Patient Characteristics and Operative Details

Only four series [16,21,25,26] reported the average ASA score (median value of 1.9, range 1.3–2.5 for SP-RADP; 1.7, range 1.4–2.3 for SP-LADP), while preoperative BMI was described in six series [16,17,19,21,25,26] (median value of 24.1, range 23.3–26.4 for SP-RADP; 24.4, range 23.4–27.3 for SP-LADP). Of the groups reporting the incidence of previous abdominal surgery [16,17,21], 5 out of 15 patients in both groups had had previous surgery in one study [20], with no patients undergoing previous surgery in the other two reports. All other patients’ characteristics are summarized in Table 1.

**Figure 3:**
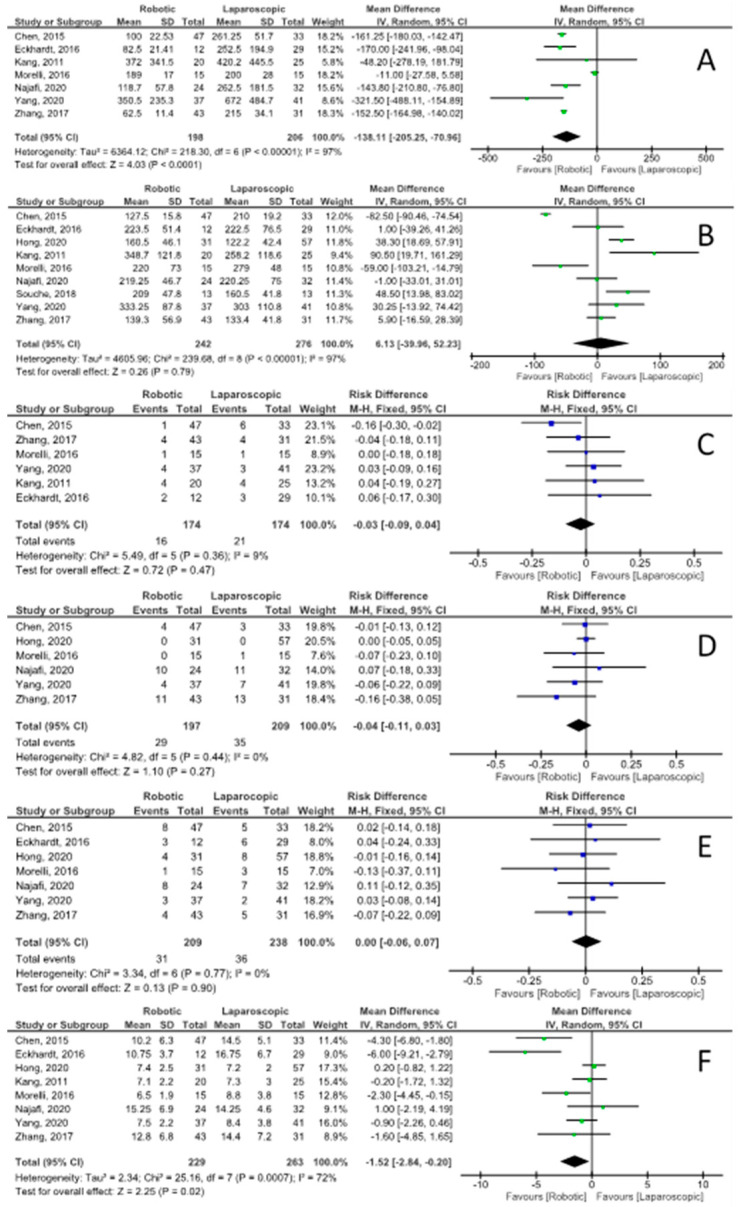
Secondary outcomes forest plots: (**A**) intraoperative blood loss (mL); (**B**) operative time (min); (**C**) perioperative blood transfusions; (**D**) Clavien–Dindo grade ≥3 complications; (**E**) postoperative pancreatic fistula grade B/C; (**F**) hospital length of stay (days).

Eight of the included studies [15,16,18,20,21,23,24,25] reported the conversion rate, with an RD of −0.05 (95% CI −0.09, −0.01) and moderate heterogeneity (I^2^ = 26%) of being converted to “open” technique favoring the robotic approach. Unfortunately, no study described the reason for conversion. The intraoperative blood loss (Figure 3), as reported in seven series [16,17,19,21,22,25,26], was significantly lower for the robotic group, with a mean difference of −138 mL (95% CI −205, −71) and high heterogeneity (I^2^ = 97%). There was no statistical difference in the operative time between the two groups (Figure 3), reported by nine series [15,16,17,18,20,21,23,24,25], with a mean difference of 6.1 min (95% CI −40, 52) and high heterogeneity (I^2^ = 97%). Four studies [17,23,24,25] reported the distal pancreatic stump closure technique, which was with an endo-GIA stapler in all cases in both groups. Eight studies [15,16,18,20,21,23,24,25] reported data on spleen preservation techniques, including a total of 211 robotic and 219 laparoscopic procedures. The Kimura technique [26] was adopted in 159 out of the 196 patients (81.1%) undergoing SP-RADP (the remaining 18.9% of patients had the pancreatic resection performed according to the technique described by Warshaw [27]) and in 84 out of the 154 SP-LADP (54.5%), with the Warshaw technique being adopted for the remaining 45.5%.

### 3.6. Postoperative Morbidity and Outcomes

Eight series [15,16,17,18,20,21,24,25] reported the perioperative mortality, with no cases of 30-day deaths. Seven studies [15,16,17,20,21,24,27] described the prevalence of POPF. The RD of clinically relevant POPF (ISGPS grade B/C) was 0.00 (95% CI −0.06, 0.07) with no heterogeneity (I^2^ = 0%). The RD of Clavien–Dindo grade ≥3 postoperative complications, as reported in six series [16,18,21,22,25,26], was −0.04 (95% CI −0.11, 0.03) with no heterogeneity (I^2^ = 0%). The mean hospital LOS difference was −1.5 days (95% CI −2.8, −0.2) in favor of SP-RADP and with high heterogeneity (I^2^ = 0%). Data on overall postoperative complications, Clavien–Dindo grade 1–2 postoperative complications, biochemical leaks, and postoperative bleeding episodes are reported in Table 3.

### 3.7. Quality of Evidence

The level of evidence was rated according to GRADE and is summarized in Table 4.

## 4. Discussion

To the best of our knowledge, this systematic review and meta-analysis is the first report summarizing all the available evidence on patients undergoing spleen-preserving distal pancreatectomy with robotic and laparoscopic techniques. All published studies comparing these two minimally invasive surgical approaches were screened in order to analyze the intention-to-treat population of patients undergoing DP where the spleen was intended to be preserved and to evaluate whether the surgical technique would have an impact on the spleen preservation success rate.

The spleen holds the largest lymphoid tissue mass in the body, producing early immunoglobulins M and containing macrophages that act as barriers against encapsulated pathogens. Avoiding unnecessary splenectomies prevents those patients undergoing DP from facing significant thromboembolic [28] and infective risks [29]. The most serious post-splenectomy complication is overwhelming post-splenectomy infection (OPSI), which can start with flu-like symptoms but can rapidly progress to septic shock, coma, and disseminated intravascular coagulation [30]. OPSI can represent a major medical emergency, with a mortality rate that can be up to 50–70% [31,32], a yearly incidence of 0.23%, and a lifetime risk of approximately 5%. The risk is greater within the first two years postoperatively but can vary depending on patient risk factors, such as age, immunological status, and indication for splenectomy [33,34]. In order to protect splenectomized individuals from such complications, prophylactic pneumococcal, Haemophilus influenzae type b, meningococcal, and annual influenza vaccinations are usually performed. Despite these risks, splenectomy is routinely performed alongside DP for pancreatic adenocarcinoma in order to achieve an adequate oncological clearance, given the high risk of lymph node involvement [35]. Spleen preservation should be considered in all patients undergoing DP for benign indications or pre-malignant/low-grade tumors, as it has been shown to be a safe procedure that can reduce perioperative morbidity and enable better long-term outcomes [36,37,38,39]. The spleen can be preserved despite the excision of the splenic vessels, as firstly described by Warshaw in 1988 [27], or with splenic vessel preservation, as demonstrated by Kimura et al. almost a decade later [26]. Both approaches have been shown to have comparable short- and long-term results in a recent international multicentric retrospective study [40] and carry fewer complications when performed with a minimally invasive technique. After early experiences of laparoscopic DP [1,2], the minimally invasive approach to pancreatic surgery has progressively gained popularity, with safety and efficacy profiles comparable to open surgery, together with reduced blood loss and a faster recovery time [41,42,43,44,45]. According to the most recent evidence-based guidelines, minimally invasive DP should be considered over open DP for all patients with benign and low-grade malignant tumors [46]. The robotic technique, with its superior accuracy, 3D vision, greater range of motion and precision [47], and excellent safety and efficacy profile in complex oncological surgery [48,49], has been utilized by several surgeons when performing pancreatic procedures [5,50,51].

This meta-analysis showed that the robotic approach is more effective than laparoscopy in allowing spleen preservation during DP, with an RD of spleen preservation failures of 0.24 (95% CI 0.15, 0.33), with reduced intraoperative blood loss (mean difference of −138 mL (95% CI −205, −71)) and similar operative time (mean difference of 6.1 min (95% CI −40, 52)). Patients undergoing SP-RADP were also less likely to experience intraoperative conversion to the “open” technique, with 3/201 open conversions (1.5%) in the robotic group and 15/219 (6.8%) in the laparoscopic group, with an RD of −0.05 (95% CI −0.09, −0.01) [15,16,18,20,21,23,24,25]. It was not possible to identify the proportion of patients where splenic vessel excision (Warshaw technique) was planned preoperatively, but a higher proportion of splenic vessel preservation was observed in patients undergoing SP-RADP (159/196 patients (81.1%)) versus SP-LADP (84/154 (54.5%)). With the exception of cases of tumor proximity or vascular involvement of the splenic vessels, when splenectomy or the Warshaw technique are usually the preferred choices, the Kimura technique is generally the preferred approach. The higher proportion of successful splenic vessel preservations in the robotic group, coupled with the superior spleen preservation rate, could reflect the more precise vascular dissection of the small tributaries of the splenic artery and vein that can be performed robotically. No differences in overall, clinically significant complications (Clavien–Dindo grade ≥3) and POPF were observed between the two groups, but patients undergoing SP-RADP had a significantly shorter hospital LOS, with a mean difference of −1.5 days (95% CI −2.8, −0.2).

Due to the lack of long-term follow-up data, the postoperative morbidity results of the present meta-analysis could underestimate the possible beneficial effects of the robotic approach in terms of expected lower incidence of complications related to the occurrence of splenic infarctions and asplenia-related infections due to the significantly higher proportion of successful splenic and splenic vessel preservation in patients undergoing SP-RADP. Prevalence of overall complications, of Clavien–Dindo grade ≥3 complications, and of clinically relevant POPF were similar to those reported in the literature following minimally invasive DP and open DP [40], with overall complications reported in 31.5% and 45.4%, Clavien–Dindo grade ≥3 complications in 14.7% and 16.7%, and clinically relevant POPF in 14.8% and 15.1% of patients undergoing SP-RADP and SP-LADP, respectively.

Unfortunately, there was no randomized controlled trial directly comparing SP-RADP and SP-LADP that could be included in the present analysis. We performed a sensitivity analysis in order to further investigate the moderate heterogeneity (I^2^ = 63%) of the main outcome.

In conclusion, both SP-RADP and SP-LADP proved to be safe and effective procedures, with minimal perioperative mortality and low postoperative morbidity. The robotic approach proved to be superior to the laparoscopic approach in terms of spleen preservation rate, intraoperative blood loss, and hospital length of stay. Future prospective and randomized studies with a longer follow-up could better evaluate the possible differences between these two techniques in terms of mid- to long-term complications and outcomes.

## Figures and Tables

**Table 1:** Summary of the selected studies with patients’ characteristics and quality assessment according to the Newcastle–Ottawa scale (NOS). NA: not available.

Author and Year	Study Type	N Rob/Lap	Age, Years Rob–Lap	Sex (F) Rob/Lap	Lesion Size, mm Rob–Lap	BMI Rob–Lap	ASA Rob–Lap	NOS Assessment		
								Selection	Comparability	Outcome
Chen et al., 2015	Matched cohort	47/33	55.6 ± 14.3–55.8 ± 16.2	31/21	31.25 ± 3.4–29 ± 3.4	24.4 ± 2.9–24.8 ± 2.7	2.5 ± 0.7–1.91 ± 0.3	3 *	2 *	3 *
Eckhardt et al, 2016	Cohort	12/29	50.5 ± 14.4–55 ± 16.8	8/17	22 ± 10.4–38 ± 3	24.00 ± 3.4–27.3 ± 4.3	NA	3 *	1 *	3 *
Hong et al, 2020	Cohort	31/57	NA	NA	36.5 ± 17.4–29.8 ± 19.5	NA	NA	3 *	1 *	3 *
Kang et al, 2011	Cohort	20/25	44.5 ± 15.9–56.5 ± 13.9	12/14	35 ± 13–30 ± 14	24.2 ± 2.9–23.4 ± 2.6	NA	3 *	1 *	3 *
Liu et al, 2017	Matched cohort	76/77	NA	NA	NA	NA	NA	3 *	2 *	3 *
Morelli et al, 2016	Case-control	15/15	58.2 ± 13.7–49.3 ± 17.1	9/13	29.9 ± 16.5–26.9 ± 13.5	26.4 ± 3.1–26.1 ± 1.9	2.40 ± 0.5–2.30 ± 0.5	2 *	2 *	3 *
Nell et al, 2016	Cohort	5/9	NA	NA	NA	NA	NA	3 *	1 *	3 *
Najafi et al, 2020	Cohort	24/32	NA	NA	NA	NA	NA	3 *	1 *	3 *
Souche et al, 2018	Cohort	13/13	NA	NA	NA	NA	NA	3 *	1 *	3 *
Yang et al, 2020	Cohort	37/41	42.9 ± 14–51.3 ± 14.6	23/27	27 ± 12–42 ± 33	23.5 ± 3.2–24.1 ± 3.4	1.41 ± 0.6–1.58 ± 0.8	3 *	1 *	3 *
Zhang et al, 2017	Cohort	43/31	47.9 ± 10.5–48.7 ± 12.3	23/19	17.5 ± 2.7–16.5 ± 2.4	23.3 ± 2.7–23.9 ± 3.2	1.26 ± 0.4–1.39 ± 0.5	3 *	1 *	3 *

**Table 2:** Sensitivity analysis by sequential omission of each individual study. Meta-analysis estimates, given the named study is omitted. CI: confidence interval.

Study Omitted	Risk Difference [95% CI] (<1 Favors Robotic)	Test of Heterogeneity		Quantification of Heterogeneity
		Chi^2^	*p*	
Chen et al, 2015	0.19 [0.13, 0.25]	10.51	0.31	df = 9; I^2^ = 14%
Eckhardt et al, 2016	0.25 [0.15, 0.35]	26.40	0.002	df = 9; I^2^ = 66%
Hong et al, 2020	0.25 [0.15, 0.36]	24.73	0.003	df = 9; I^2^ = 64%
Kang et al, 2011	0.23 [0.14, 0.33]	26.49	0.002	df = 9; I^2^ = 66%
Liu et al, 2017	0.25 [0.15, 0.36]	27.61	0.001	df = 9; I^2^ = 67%
Morelli et al, 2016	0.24 [0.14, 0.34]	27.11	0.001	df = 9; I^2^ = 67%
Najafi et al, 2020	0.25 [0.16, 0.35]	24.78	0.003	df = 9; I^2^ = 64%
Nell et al, 2016	0.23 [0.13, 0.32]	24.15	0.004	df = 9; I^2^ = 63%
Souche et al, 2018	0.26 [0.16, 0.25]	24.49	0.004	df = 9; I^2^ = 63%
Yang et al, 2020	0.24 [0.14, 0.34]	27.30	0.001	df = 9; I^2^ = 67%
Zhang et al, 2017	0.23 [0.14, 0.33]	26.34	0.002	df = 9; I^2^ = 66%

**Table 3:** Risk differences between robotic and laparoscopic spleen-preserving distal pancreatectomies. CI: confidence interval; POPF: postoperative pancreatic fistula.

Outcome	Studies	Risk Difference [95% CI] (<1 Favors Robotic)	Test of Heterogeneity		Quantification of Heterogeneity
			Chi^2^	*p*	
Spleen preserving failure	16–26	−0.25 [−0.30, −0.19]	27.22	0.002	df = 10; I^2^ = 63%
Open conversions	16, 17, 19, 21, 22, 24–26	−0.05 [−0.09, −0.01]	9.41	0.22	df = 7; I^2^ = 26%
Overall complications	16–19, 21, 25, 26	−0.06 [−0.14, 0.02]	2.15	0.91	df = 6; I^2^ = 0%
Complications—Clavien–Dindo grade 1–2	16, 18, 21	−0.02 [−0.15, 0.11]	1.00	0.61	df = 2; I^2^ = 0%
Complications—Clavien–Dindo grade ≥3	16, 18, 21, 22, 25, 26	−0.04 [−0.11, 0.03]	4.82	0.44	df = 5; I^2^ = 0%
POPF grade B/C	16–18, 21, 22, 25, 26	0.00 [−0.06, 0.07]	3.34	0.77	df = 6; I^2^ = 0%
Biochemical leaks	16–18, 21, 26	−0.04 [−0.14, 0.05]	1.01	0.91	df = 4; I^2^ = 0%
Intra-/post-operative blood transfusions	16, 17, 19, 21, 25, 26	−0.03 [−0.09, 0.04]	5.49	0.36	df = 5; I^2^ = 9%
Reoperation rate	16, 17, 21, 22, 26	0.01 [−0.05, 0.07]	3.86	0.42	df = 4; I^2^ = 0%
Hospital length of stay	16–19, 21, 22, 25, 26	−1.52 [−2.84, −0.20]	25.16	<0.001	df = 7; I^2^ = 72%

**Table 4:** Robotic versus laparoscopic surgery for spleen-preserving distal pancreatectomies. * The risk in the intervention group (and its 95% confidence interval) is based on the assumed risk in the comparison group and the relative effect of the intervention (and its 95% CI). CI: Confidence interval; RR: Risk ratio; MD: Mean difference.

Outcomes	N of Participants (Studies) Follow up	Certainty of the Evidence (GRADE)	Relative Effect (95% CI)	Anticipated Absolute Effects	
				Risk with Laparoscopic Approach	Risk Difference with Robotic Approach
Spleen preservation rate	685 (11 observational studies)	⨁⨁◯◯ LOW	RR 1.31 (1.16 to 1.48)	680 per 1000	211 more per 1000 (109 more to 326 more)
Blood Loss	404 (7 observational studies)	⨁⨁◯◯ LOW	-	Mean blood loss was 233.3 mL	MD 138.11 lower (205.25 lower to 70.96 lower)
Operative time	518 (9 observational studies)	⨁⨁◯◯ LOW	-	Mean operative time was 206.1 min	MD 6.13 higher (39.96 lower to 52.23 higher)
Pancreatic fistula grade B–C	447 (7 observational studies)	⨁⨁◯◯ LOW	RR 1.03 (0.66 to 1.60)	151 per 1000	5 more per 1000 (51 fewer to 91 more)
Complications Clavien–Dindo 3–4	406 (6 observational studies)	⨁⨁◯◯ LOW	RR 0.79 (0.52 to 1.20)	167 per 1000	35 fewer per 1000 (80 fewer to 33 more)
Hospital length of stay	492 (8 observational studies)	⨁⨁◯◯ LOW	-	Mean hospital stay was 9.8 days	MD 1.52 lower (2.84 lower to 0.2 lower)
Perioperative bleeding	143 (3 observational studies)	⨁⨁◯◯ LOW	RR 0.93 (0.24 to 3.63)	55 per 1000	4 fewer per 1000 (42 fewer to 144 more)

## Data Availability

The data used for this manuscript are available upon request of the reviewers.

## Abbreviations

ASA:	American Society of Anesthesiologists
BMI:	body mass index
CI:	confidence interval
DP:	distal pancreatectomy
IPMN:	intraductal papillary mucinous neoplasm
LOS:	length of stay
NET:	neuroendocrine tumors
OPSI:	overwhelming post-splenectomy infection
POPF:	postoperative pancreatic fistula
RD:	risk difference
SP-LADP:	spleen-preserving laparoscopic-assisted distal pancreatectomy
SP-RADP:	spleen-preserving robot-assisted distal pancreatectomy
